# Hyperopic refractive correction by LASIK, SMILE or lenticule reimplantation in a non-human primate model

**DOI:** 10.1371/journal.pone.0194209

**Published:** 2018-03-28

**Authors:** Geraint P. Williams, Benjamin Wu, Yu Chi Liu, Ericia Teo, Chan L. Nyein, Gary Peh, Donald T. Tan, Jodhbir S. Mehta

**Affiliations:** 1 Tissue Engineering and Stem Cell Group, Singapore Eye Research Institute, Singapore; 2 Singapore National Eye Centre, Singapore; 3 Ophthalmology Academic Clinical Program, Duke-NUS Graduate Medical School, Singapore; 4 Department of Ophthalmology, Yong Loo Lin School of Medicine, National University of Singapore, Singapore; 5 Department of Clinical Sciences, Duke-NUS Graduate Medical School, Singapore; Oklahoma State University Center for Health Sciences, UNITED STATES

## Abstract

Hyperopia is a common refractive error, apparent in 25% of Europeans. Treatments include spectacles, contact lenses, laser interventions and surgery including implantable contact lenses and lens extraction. Laser treatment offers an expedient and reliable means of correcting ametropia. LASIK is well-established however SMILE (small-incision lenticule extraction) or lenticule implantation (derived from myopic laser-correction) are newer options. In this study we compared the outcomes of hyperopic LASIK, SMILE and lenticule re-implantation in a primate model at +2D/+4D treatment. While re-implantation showed the greatest regression, broadly comparable refractive results were seen at 3-months with SMILE and LASIK (<1.4D of intended), but a greater tendency to regression in +2D lenticule reimplantation. Central corneal thickness showed greater variation at +2D treatment, but central thickening during lenticule reimplantation at +4D treatment was seen (-17± 27μm LASIK, -45 ± 18μm SMILE and 28 ± 17μm Re-implantation; p <0.01) with expected paracentral thinning following SMILE. Although in vivo confocal microscopy appeared to show higher reflectivity in all +4D treatment groups, there were minimal and inconsistent changes in inflammatory responses between modalities. SMILE and lenticule re-implantation may represent a safe and viable method for treating hyperopia, but further optimization for lower hyperopic treatments is warranted.

## Introduction

Clear vision depends on the cornea and lens focusing light from a distance object on to the retina and is termed emmetropia. Refractive errors (ametropia) can broadly be divided in to hyperopia where light is focused behind the retina in the unaccommodated eye, myopia where light convergence occurs in front of the retina or astigmatism where light passes through the cornea or lens at different meridia resulting in the formation of focal lines as opposed to a focal point. Ametropia is common and affects half of European adults with a prevalence of hyperopia (also called long/far-sightedness) of 25%. [1] There is considerable ethnic variation however with a higher prevalence of hyperopia among White compared to Afro-Caribbean and Asian children. [2] [3]

**Fig 1:**
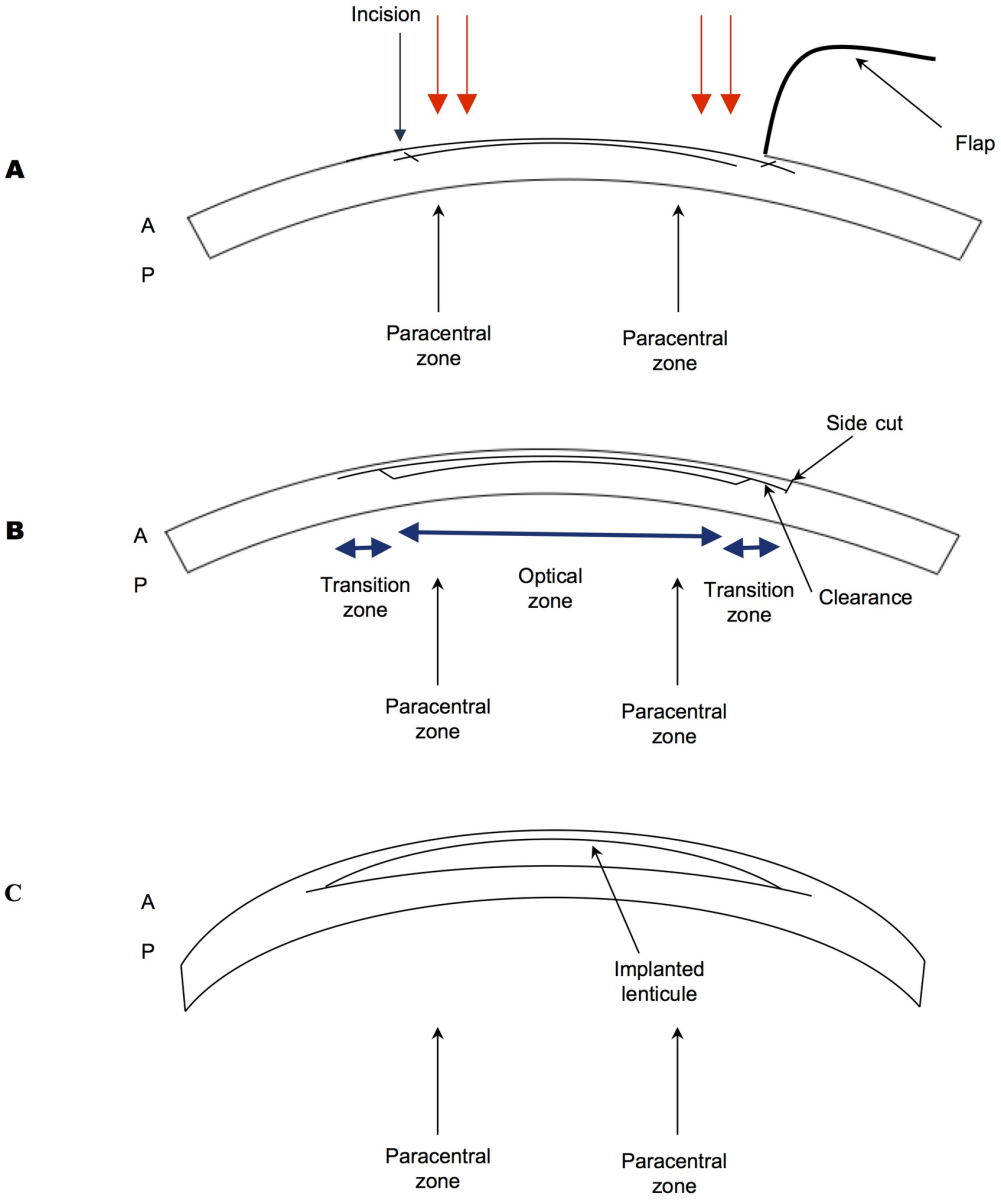
Cartoon schematic of hyperopic treatments. A, LASIK: Corneal is lifted, excimer laser (represented by red arrows) is applied to the peripheral cornea in a circular pattern. B, SMILE lateral view of cornea showing optical (anterior cap) zone (5.5mm diameter), the transition zone (1mm wide) and the 90 degrees lenticule side cut C, Lenticule shape: Showing the implanted lenticule. A = Anterior Cornea, P = Posterior Cornea.

Hyperopia may be corrected by wearing spectacle or contact lenses, or treated with surgical procedures such as implantable contact lenses or clear lens extraction. [4, 5] Laser refractive treatment offers a definitive treatment without the inherent risks of intra-ocular surgery. Photorefractive keratectomy (PRK) and Laser Assisted In Situ Keratomileusis (LASIK) are common well-established techniques for correcting hyperopia[6]. The latter involves the creation of a hinged corneal flap, and an excimer laser is then applied to re-sculpt the corneal stroma into a steeper configuration (Fig 1A), in the former the excimer laser ablation takes place after the removal of corneal epithelium without the need for flap. LASIK flaps may be created by a microkeratome or a femtosecond laser; the latter offers a more controlled, predictable means of creating corneal flaps. [7–9]

A recent advancement involves all in one femtosecond laser refractive procedure, refractive lenticule extraction (ReLEx), which circumvents the requirement for an excimer laser. (Fig 1B) [10–12]. In small-incision lenticule extraction (SMILE), a small incision version of ReLEx, a 2–4mm ‘key-hole’ incision is made to access the lenticule without the need to create a full flap [13, 14]. Experimental data from animal models suggests faster nerve recovery following SMILE and clinical studies have supported these findings and demonstrated lower incidence of dry eye problems. [15, 16] Data has also shown comparable visual results to LASIK for myopic correction. [17, 18].

Hyperopic correction presents a different challenge to myopic correction, as regression of refractive power is a common issue post-LASIK [19] SMILE was originally only licensed for myopic and myopic/astigmatism corrections. However newer software enhancements have made hyperopic treatment possible. Hyperopic treatment in combination with ReLEx can be achieved in two ways: 1) by hyperopic smile laser lenticule creation, or 2) by lenticule re-implantation [20].

In hyperopic SMILE treatment, the femtosecond laser dissects out a concave lenticule within the cornea stroma, which is then extracted by the surgeon, achieving the end result of a steeper central corneal configuration. The second option to correct hyperopia is through convex-shaped lenticule re-implantation, which has been obtained from a myopic correction in another eye (Fig 1C). The latter has been shown to be efficacious in both human and primates. [21–24] Previously in our laboratory, we have demonstrated the feasibility of re-implantation of refractive lenticules in a rabbit model [25, 26] and non-human primate model, with minimal short-term corneal haze and wound healing responses [20, 26].

There is limited published clinical outcome data on the use of SMILE to correct hyperopia. [24, 27] We have also previously showed in a rabbit model that hyperopic LASIK induces more inflammation than SMILE, albeit at higher treatment profiles in the early post-operative period. [28] Comparable data for hyperopic LASIK, SMILE and lenticule reimplantation to our knowledge does not exist. As non-human primates share the greatest genetic homology with humans (including the presence of a corneal Bowman’s layer), a monkey model afforded a means of assessing the inflammatory effects of all three treatment modalities prior to applying the technology in human patients [25, 26]. The purpose of this study therefore was to determine and compare the refractive outcomes and corneal inflammatory responses of hyperopic LASIK, SMILE and lenticule re-implantation in a non-human primate model at +2.0D and +4.0D treatments.

## Materials and methods

### Non-human primate model

18 healthy non-human primates, Macaca fascicularis (cynomolgus macaque), aged 2 to 5 years and weighing between 3 to 5 kg, obtained from Nafovanny, Vietnam, were used in this study. All experiments were specifically approved and performed in accordance with relevant guidelines and regulations set out by and approved by SingHealth, Singapore. The use of animals in this study were specifically approved and adhered to the SingHealth Institutional Animal Care and Use Committee (IACUC) protocol 2014/SHS/ 1011 and conformed to the ARVO Statement for the Use of Animals in Ophthalmic and Visual Research. One monkey suffered from microbial keratitis following +4 SMILE treatment, the results of which were excluded. Those data points were replaced with repeat experiments on a replacement monkey (n = 1) with the fellow untreated eye serving as a negative control.

Macaques were randomly divided into six groups of three monkeys. In the first two groups, +2.0D hyperopic treatments were performed, with the LASIK procedure on one eye of each monkey, and the SMILE procedure on the other eye (n = 6). In the next two groups, +4.0D hyperopic treatments were performed, with LASIK on one eye of each monkey, and SMILE on the other eye (n = 6). In the fifth group, a -2.0D lenticule was extracted from one eye of each monkey and implanted autologously into the other eye (n = 3). In the sixth group, a -4.0D lenticule was extracted from one eye each monkey and implanted autologously into the fellow eye (n = 3) (S1 Fig).

During the procedures, the monkeys were tranquilized intramuscularly with Ketamine Hydrochloride 10mg/kg or Medetomidine 0.02mg/kg. Anaesthesia was induced with 2–3% inhaled isoflurane and maintained with 1–2% inhaled isoflurane.

In the event that pain was observed at any time, such as when there was difficulty in opening of the eyes, withdrawal or protection of the eyes, rubbing or scratching of the eyes, decrease in food or water intake, or awkward or abnormal postures, Pentazocine analgesia 3-4mg/kg was given intramuscularly.

### LASIK procedure

LASIK flaps were created with the VisuMax femtosecond laser system (Carl Zeiss Meditec, Jena, Germany). The corneal flaps were lifted, and a 7.5-mm optical zone ablation was performed using an excimer laser (Technolas; Bausch & Lomb, New Jersey, U.S.A.) [28]. Fig 1A. The flaps were subsequently repositioned, and two interrupted sutures were used to hold the flap in place (10–0 Nylon). A bandage contact lens was immediately applied, and temporary tarsorrhaphy was used to close the eyelids. A subconjunctival injection of dexamethasone and gentamicin was given and repeated at post-operative day 3 at the time of suture removal. After surgery, 0.3% Tobramycin and 0.1% Dexamethasone eye drops were administrated four times daily for 1 week.

### SMILE procedure

SMILE was performed using a VisuMax femtosecond laser system (VisuMax, Carl Zeiss Meditec, Jena, Germany). A small interface cone was used in all of the procedures. A special nomogram was used to calculate the geometry and the thickness of the refractive lenticule for the correction of hyperopia. The femtosecond laser parameters used in this experiment were: 120μm flap thickness, 7.9mm flap diameter, 170nJ power, and side cut angles at 90 degrees. The spot distance and tracking spacing were set respectively at 3μm, 3μm for the lenticule, 1μm, 1μm for the lenticule side, 3μm, 3μm for the flap, and 2μm, 2μm for the flap side cut. The diameter of the lenticule was 7.5mm, which is equal to the optical zone (5.5mm) plus the transition zone (1mm + 1mm). Once suction was applied, laser incisions were made in the following automated sequence: 1) a spiral-in pattern on the posterior surface of the lenticule with a 5.5mm diameter, equating the optical zone, 2) a spiral-out pattern on the posterior surface of the lenticule, from the edge of the optical zone for 2.0mm, corresponding to the transition zone, 3) a vertical 90 degree lenticule side cuts, 4) a spiral-out anterior surface of the lenticule, cutting 7.9mm diameter cap 5) followed by a superiorly placed 2.5mm wide incision at 120 degrees as previously optimised [26, 28] Fig 1B and 1C. After the laser sequence was completed, a Seibel spatula (Rhein Medical Inc., Petersburg, Florida, U.S.A.) was inserted into the superiorly placed 3mm incision to gain access to the intrastromal lenticule. Following which, a SMILE dissector was introduced to dissect microadhesions—firstly on the anterior and then the posterior surfaces of the lenticule from the surrounding stroma. Once these planes were separated, the lenticule was extracted using a co-axial Tan DSAEK forceps (ASICO, Illinois, U.S.A.). The cornea stromal pocket was then irrigated with balanced salt solution via a 25-gauge cannula. A subconjunctival injection of dexamethasone and gentamicin was given and repeated at post-operative day 3 at the time of suture removal. After surgery, 0.3% Tobramycin and 0.1% Dexamethasone eye drops were administrated four times daily for 1 week.

### Lenticule re-implantation procedure

Lenticule extraction was performed using myopic SMILE: a spiral-in pattern for the posterior surface of the lenticule, spiral-out pattern for the anterior surface of the lenticule, followed by a superiorly placed 3mm wide incision at 120 degrees. 160μm deep stromal pockets were created using VisuMax femtosecond laser system (Carl Zeiss Meditec, Jena, Germany), with a 7.9mm diameter spiral-in lamellar cut, followed by a 90 degree side cut, through which the extracted lenticule is implanted. [25] [26]. This allowed reproducible formation of pockets for re-implantation of lenticules. Closure of the side-cut with two 10–0 nylon sutures was done to ensure secure the implantation of the lenticule and removed at 72 hours. A subconjunctival injection of dexamethasone and gentamicin was given and repeated at post-operative day 3 at the time of suture removal. After surgery, 0.3% Tobramycin and 0.1% Dexamethasone eye drops were administrated four times daily for 1 week.

### Slit-lamp photography

Slit-lamp photographs were taken with a Zoom Slit Lamp NS-2D (Righton, Tokyo, Japan). Images were collected pre-operation and post-operation (at 1 week, 1 month and 3 months).

### Anterior segment optical coherence tomography

Corneal cross-sectional visualization and measurement of corneal thickness were performed using RTVue Spectral Domain Optical Coherence Tomography (Optovue, Fremont, California, U.S.A.). The examiner adjusted the system to position the vertex at the centre of the AS-OCT image and then slowly moved the system away from the cornea until the vertical white beam was barely seen. Images were taken pre-operation and post-operation (at 1 week, 1 month and 3 months). Measurements were para-centrally taken at 4mm diameter (2mm from the centre of the cornea).

### Spherical error measurement

Spherical errors were measured pre-operatively and post-operatively on 1 week, 1 month, 3 months. Measurements were taken using a Nidek ARK-30 Autorefractor (Hiroishi, Japan).

### Corneal topography and corneal curvature

Measurements of corneal topography and corneal curvature (keratometry) were obtained pre-operation and post-operation, at 1 day, 1 week, 1 month and 3 months. Measurements were taken using the ATLAS 9000 Corneal Topography System (Carl Zeiss Meditec, Jena, Germany).

### In-vivo confocal microscopy

In-vivo confocal microscopy was performed pre-operation, and post-operatively on 1 week, 1 month and 3 months for visualization of the corneal stroma cells. This was performed using a Heidelberg retinal tomography HRT3 with Rostock corneal module (Heidelberg Engineering GmbH, Heidelberg, Germany). A carbomer gel (Vidisic; Mann Pharma, Berlin, Germany) was used as immersion fluid. All corneas were examined centrally from epithelium to endothelium and the LASIK, SMILE or re-implantation interface identified. In-vivo confocal micrographs were then analysed with the Heidelberg Eye Explorer version 1.5.1 software (Heidelberg Engineering GmbH, Heidelberg, Germany). Three micrographs of the flap interface (LASIK group) or lenticule plane (SMILE and re-implantation groups) were selected and analysed by mean grey value quantification of reflectivity using Image J (http://imagej.nih.gov/ij/; provided in the public domain by the National Institutes of Health, Bethesda, MD, USA) [28, 29] and keratocyte density (cells/image).

### Immunohistochemistry

After euthanisation at 3 months, the corneas were excised from the globe and embedded in Optimal Cutting Temperature (OCT) cryo-compound (Leica Microsystems, Nussloch, Germany). Frozen tissue blocks were stored at −80°C until sectioning. Serial sagittal corneal 7μm sections were cut using a cryostat (Microm HM 525, Walldorf, Germany). Sections were placed on polylysine-coated glass slides and air dried for 15 minutes.

Immunohistochemical staining was performed with sections fixed with 4% paraformaldehyde (Sigma) for 15 minutes, then washed with 1X PBS and blocked in 1X PBS with 0.15% Saponin (Sigma), 0.1% bovine serum albumin (Sigma) and 1.5% Normal Goat Serum Invitrogen) for 10 minutes. The sections were subsequently incubated with the following primary antibodies diluted in primary antibody diluent (1X PBS, 0.425% saponin, 1% BSA, 1.5% NGS, 0.0015% tween-20, 0.0015% triton-X): mouse monoclonal antibody against cellular fibronectin (Millipore, Billerica, Massachusetts, U.S.A.) diluted 1: 100; tenascin-C (Abcam, Cambridge, U.K.) diluted 1: 200; mouse monoclonal antibody against heatshock protein 47 (Hsp47) (Enzo Lifesciences, Switzerland) diluted 1:200; collagen type I (Sigma) diluted 1: 100; and CD18 (Novus Biologicals, Littleton, Colorado, U.S.A.) diluted 1: 100 in the blocking solution. After washing with 1X PBS, the sections were incubated with goat anti-mouse Alexa Fluor 488-conjugated secondary antibody (Invitrogen) at room temperature for 1 hour. Slides were then mounted with UltraCruz Mounting Medium containing DAPI (Santa Cruz Biotechnology) and were observed and imaged with a Zeiss AxioImager Z1 fluorescence microscope (Carl Zeiss, Oberkochen, Germany).

Double immunohistochemical staining was performed with sections fixed with 4% paraformaldehyde (Sigma) for 15 minutes, then washed with 1X PBS and blocked in 1X PBS with 0.15% Saponin (Sigma), 0.1% bovine serum albumin (Sigma) and 1.5% Normal Goat Serum Invitrogen) for 10 minutes. Tissue sections were incubated with mouse monoclonal antibody against α-smooth muscle actin (α-SMA, Dako Cytomation, Glostrup, Denmark) diluted 1: 50 in the blocking solution, or with mouse monoclonal antibody against Ki-67 diluted 1:200 in the blocking solution (Invitrogen, Carlsbad, CA) at 4°C overnight. On the following day, the sections were incubated with Alexa Fluor 546-conjugated secondary antibody (Invitrogen) at room temperature for 1 hour. After washing with 1X PBS, the sections were double stained with an Alexa Fluor 488-conjugated phalloidin probe (Invitrogen) at room temperature for 30 minutes. Slides were subsequently mounted with UltraCruz Mounting Medium containing DAPI (Santa Cruz Biotechnology) and observed with the Zeiss AxioImager Z1 microscope (Carl Zeiss).

To detect apoptotic cells, a fluorescence-based TUNEL assay (In Situ Cell Death Detection Kit, Roche Applied Science, Indianapolis, Indiana, U.S.A.) was used according to the manufacturer’s instructions. Counts were related to the number of DAPI positive cells to derive a percentage.

### Statistical analysis

All statistical analyses were performed using Graph Pad Prism 6 for Macintosh (La Jolla, California, U.S.A.). Results were expressed as mean ± standard deviation. The p-values were determined using the two-tailed Student's t-test for groups of two, and one-way ANOVA for comparing means of three groups or more with Bonferroni’s post-hoc correction to compare individual groups. Significant was defined as p < 0.05.

## Results

### Slit-lamp biomicroscopy and anterior segment Optical Coherence Tomography (OCT) assessment

**Fig 2:**
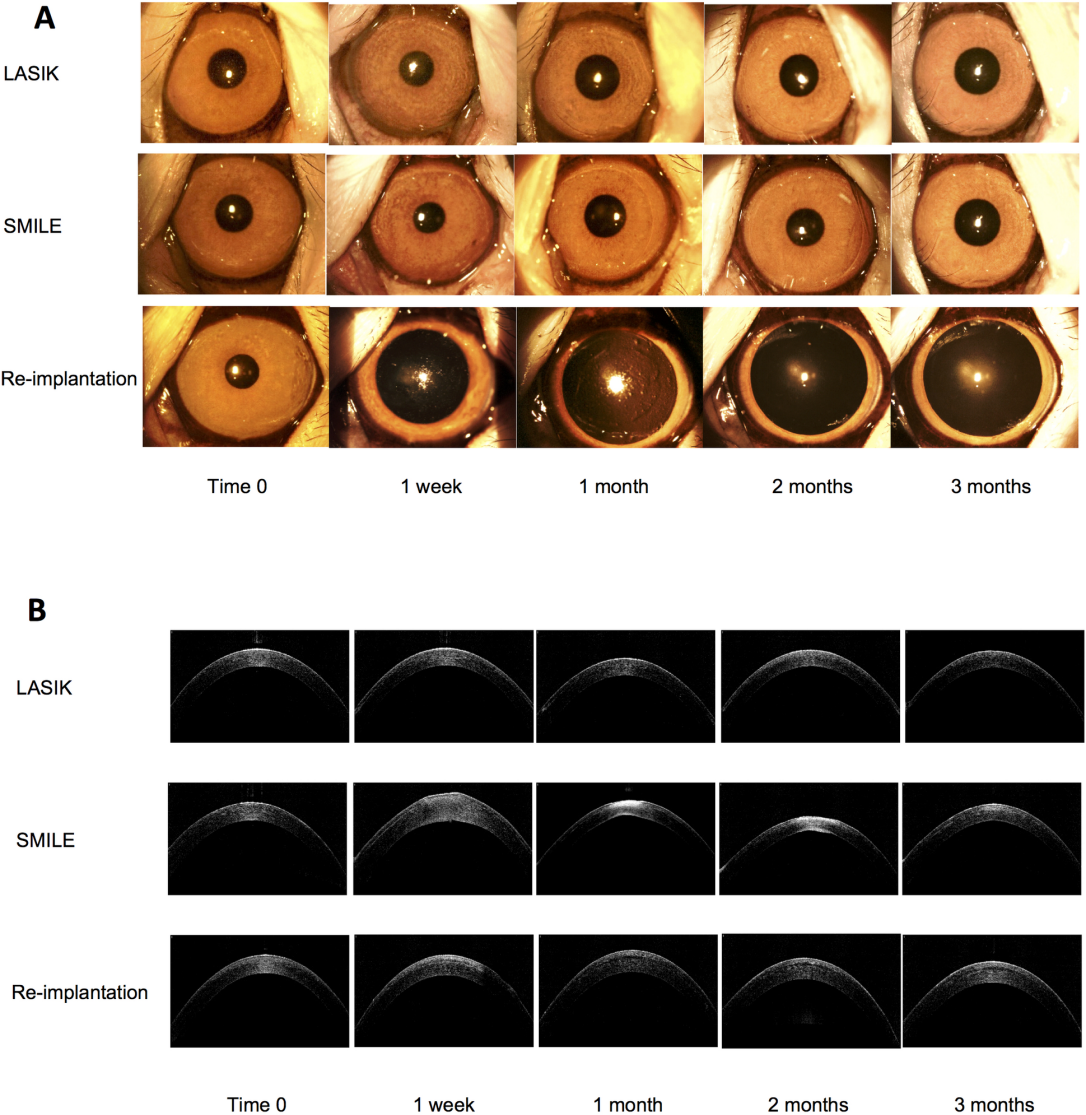
Representative colour photographs of macaque eyes before and following treatment by hyperopic LASIK, SMILE and re-implantation. Panel A show representative colour photographs of the macaque eyes before and at incremental time points following three hyperopic treatments. Corresponding AS-OCT (RTVue) images of the same time points are shown in Panel B.

Clear corneas were seen as early as one-week post treatment in all three treatment groups at +2 and +4D of treatment (Fig 2A). There was no subjective opacity for any time point as early as 1 week post-procedure (Fig 2A). OCT imaging demonstrated the effects of corneal steepening by LASIK, SMILE and lenticule re-implantation with representative OCT images derived from +4 treatment groups over the course of 3 months (Fig 2B). In order to compare steepening, the central and paracentral thickness was measured by AS-OCT (see Fig 1 for schematic diagrams). For central thickness, in the +2.0D treatment groups, there was a significant difference in the mean delta of the central corneal thickness at 3 months (14 ± 19μm LASIK, -18 ± 21μm SMILE and 16 ± 31μm Re-implantation; p < 0.05). In the +4.0D treatment groups, the mean difference in central corneal thickness was -4.7 ± 15μm, -30 ± 9.7μm and -9 ± 68μm respectively (p = 0.36) at 3 months. For paracentral changes, in the +2.0D treatment groups, there was no significant difference in the mean delta of the paracentral corneal thickness at 3 months (5.5± 30μm LASIK, -36 ± 33μm SMILE and -7 ± 29μm Re-implantation; p = 0.05). In the +4.0D treatment groups however, there was a significant difference in the mean delta of the paracentral corneal thickness at 3 months between groups (-17± 27μm LASIK, -45 ± 18μm SMILE and 28 ± 17μm Re-implantation; p <0.01).

### Intra-ocular pressure

Intra-operative pressure measurements taken over 3 months are summarised in Table A in S1 File.

### Refractive error and topography

**Fig 3:**
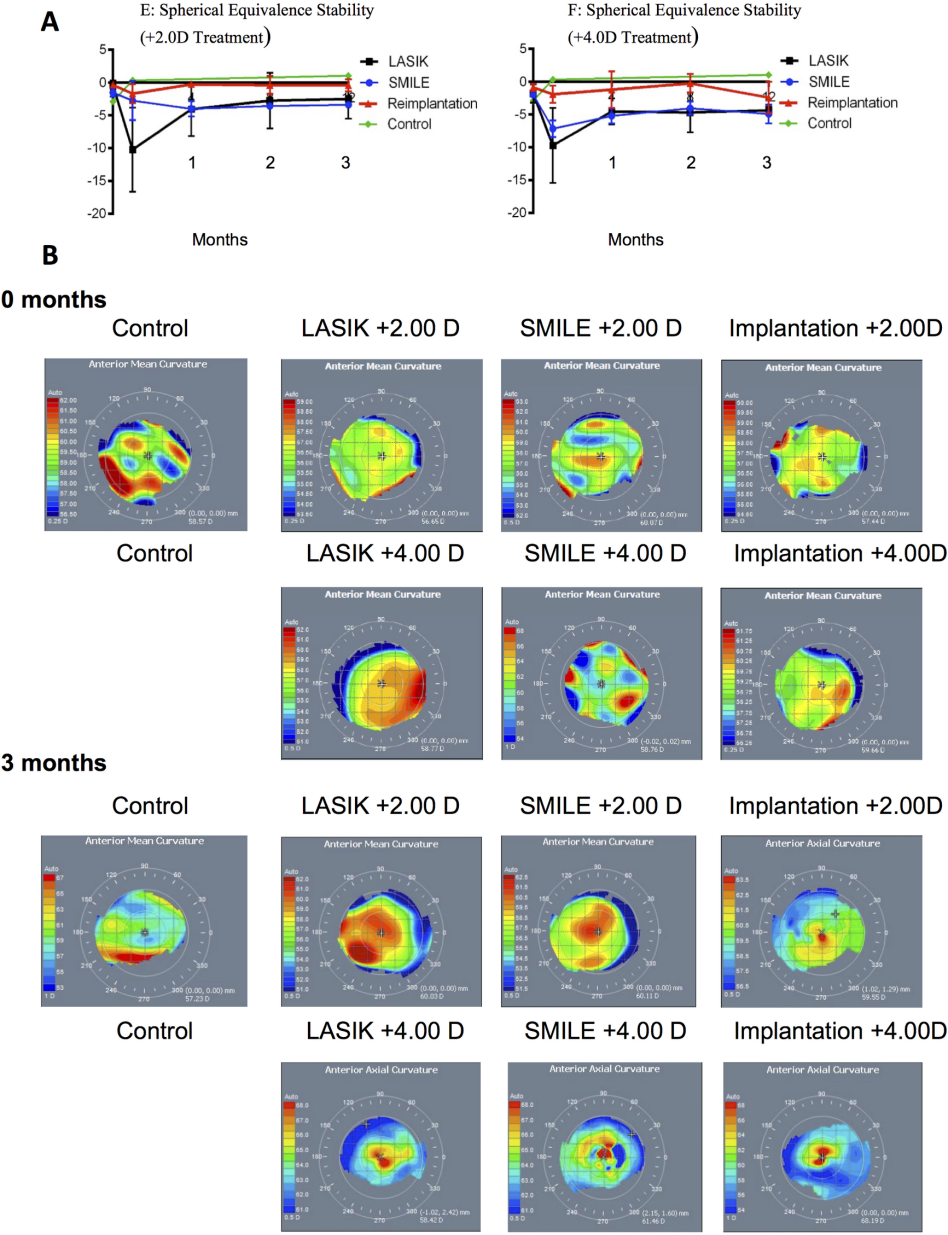
Timeline of refractive error and topographic anterior curvature at 3 months post hypermetropic correction. Panel A shows a timeline for +2 and +4 treatment groups by mean spherical equivalence (SD) for LASIK (black, n = 6)), SMILE (blue, n = 6), lenticule re-implantation (red, n = 3) and control (green, n = 1). Panel B shows representative topographical maps of the anterior curvature of the cornea (ATLAS 9000 Corneal Topography System) at 3 months post-treatment in all treatment groups.

For the +2.0D treatment group, the pre-operative mean spherical equivalent was -0.37 (SD±0.66D), -0.99 (SD±0.97D) and -0.36 (SD±0.13D) in the LASIK, SMILE and re-implantation eyes respectively, with a change of -2.5 (SD±3.0D), -3.40 (SD±1.20D) and -0.40 (SD±1.6D) at 3 months (Fig 3A). Although not statistically significant, there was a trend to regression in the re-implantation group with regards to stability over 3 months (Fig 3A).

For the +4.0D treatment group, a pre-operative mean spherical equivalent was -1.8 (SD±1.4D), -2.0 (SD±1.3D) and -0.86 (SD±0.94D) in the LASIK, SMILE and re-implantation group. The postoperative mean spherical equivalent changed to -4.4 ± 0.58D (range: -3.7 to -5.1D), -4.90 ± 3.60D (range: 0.92 to -9.4D) and -2.40 ± 4.2D (range: 2.3 to -5.6D) in the LASIK, SMILE and re-implantation eyes respectively. Stability is represented in Fig 3A.

Although there was no significant difference in the keratometry measurements following surgery, a trend towards steepening of the cornea consistent with intended hyperopic treatment was seen (Fig 3B). For the +2.0D group, the mean 3-month changes were 0.78 ± 2.1D, 1.3 ± 1.5D and 1.4 ± 1.8 D for LASIK, SMILE and re-implantation (p = 0.85) and 1.2 ± 1.2D, 1.5 ± 2.6D and 0.90 ± 2.3 D for the +4.0D group (p = 0.9). Full details are shown in Table B and C in S1 File including the untreated eye of one macaque.

### In vivo and ex-vivo inflammatory responses

**Fig 4:**
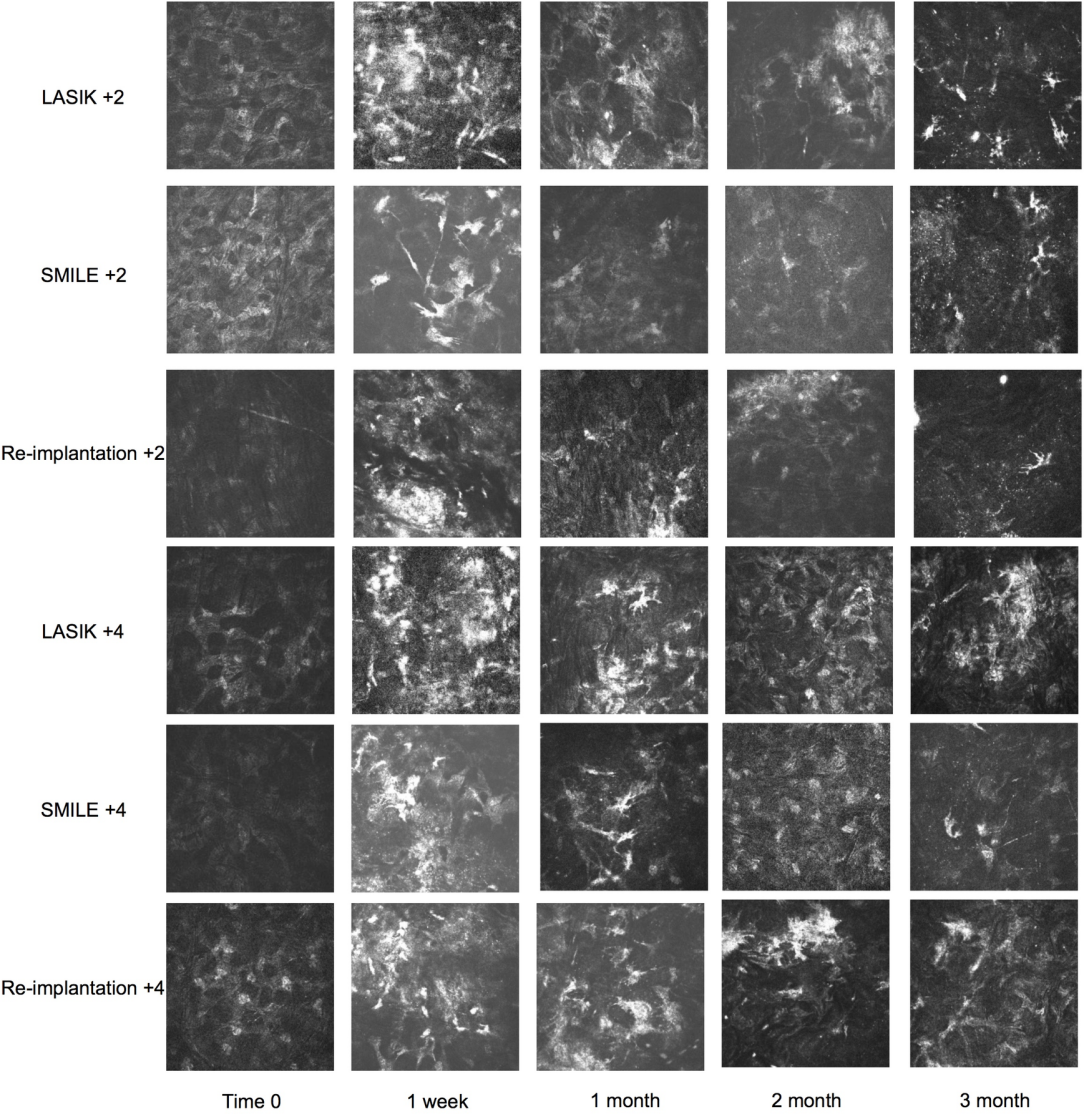
In-vivo confocal microscopy images on section plane with different hypermetropic treatments. Panel A shows in vivo confocal images taken at the femtosecond laser interface at time zero through 3 months with LASIK +2.0D, SMILE +2.0D, Re-implantation +2.0D and LASIK +4.0D, SMILE +4.0D, Re-implantation +4.0D (top to bottom). Panel B shows the delta interface reflectivity (reflectivity at 3 months-reflectivity at time 0). Panel C shows the delta interface keratocyte density (density at 3 months-density at time 0). Comparisons were made with ANOVA with Bonferroni’s post hoc test; *** = p<0.001. NS = Not Significant.

Real-time in vivo imaging of the treatment interface demonstrated hyper-reflective particles, most pronounced on week 1 post-operation by all laser approaches (Fig 4A). Hyper-reflectivity (secondary to activated keratocytes) can be seen at one month post-operation but gradually subsided by 3 months. Delta reflectivity (reflectivity at 3 months minus baseline) at the flap interface (LASIK group) or lenticule plane (SMILE and re-implantation groups) demonstrate an overall difference between groups (p<0.001) due to higher reflectivity in LASIK, SMILE and reimplantation at +4 compared to +2 (Fig 4B; p<0.001 for all comparisons). No differences were seen between LASIK vs. SMILE, LASIK vs. reimplantation or SMILE vs. reimplantation at +2D treatment (p = NS) or at +4D (p = NS). No difference in keratocyte density was observed at 3 months in all groups (p = 0.14, Fig 4C) compared to presentation (9 cells/image for LASIK +2, 10 cells/ image SMILE +2, 6.2 cells/ image lenticule reimplantation +2, 8.4 cells/image for LASIK +4, 8.5 cells/image for SMILE +4, 7.8 cells/imagelenticule reimplantation +4 at presentation). These findings were substantiated by immunohistochemical assay.

**Fig 5:**
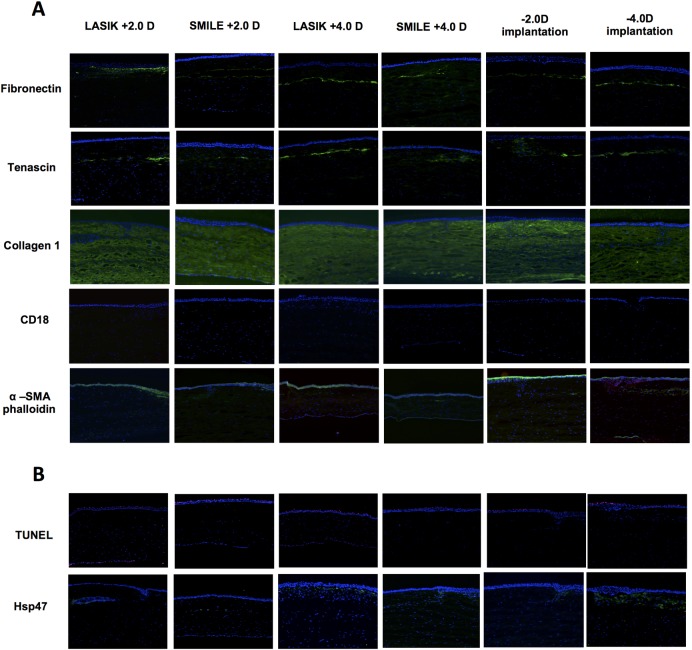
Immunohistochemical analysis of macaque corneas. Representative images of staining at 3 months post treatment are shown from the central cornea. Staining was undertaken with Fibronectin (green), Tenacsin (green), Collagen I (green), CD18, α-SMA(red)-phalloidin (green) (panel A) and HSP 47 (green), TUNEL (red) and Ki67 (green) (panel B) and shown (from left to right) for the following treatment groups: LASIK +2.0D, SMILE +2.0D, LASIK +4.0D, SMILE +4.0D, re-implantation +2.0D, re-implantation +4.0D.

Fig 5A demonstrates the immunohistochemical staining undertaken at 3 months following procedure in all groups. Fibronectin and Tenascin, markers for inflammation and injury can be seen in the plane of incision in all groups (+2.0D and +4.0D LASIK, SMILE and re-implantation) with variable expression. The dominant corneal collagen, Collagen type I, was uniformly expressed throughout the stroma in all post-operative cornea samples and showed no alteration between groups. Leukocyte integrin β2, or CD18, was not found in any of the samples by the third month post-operation. Myofibroblasts, shown by α-SMA staining, were also absent in all the samples by 3 months post-operation. Phalloidin was distributed at a subepithelial level at a variable intensity across groups.

Apoptotic TUNEL and HSP 47 (collagen-binding protein) positive cells were found within the lenticule or stroma of all cornea samples (Fig 5B, Table D in S1 File). Significant differences were seen between TUNEL positive cells with the percentage of TUNEL positive cells were increased at a higher refractive treatment for LASIK (8.6% [SD0.4] for +2 and 11.8% [SD1.0] for +4; p<0.001). While a similar trend was seen for reimplantation (8.2% [SD0.2] for +2 and 9.6% [SD0.13] for +4; p = NS) and a reverse trend for SMILE (10.9% [SD0.6] for +2 and 9.9 [SD0.2] for +4; p = NS). This also reflected a higher number of TUNEL positive cells for SMILE at +2 treatment compared to LASIK or reimplantation (p<0.001) but again this was reversed at +4 treatment, with a higher percentage seen with LASIK (p<0.05). An increase in HSP47 cells was observed in the +2 (33 [SD0.3] positive cells/field for +2 LASIK, 17 [SD1] +2 SMILE, 19 [SD 1] +2 reimplantation and 29%;p<0.01) and +4 treatment groups (38 [SD1.7] positive cells/field for +4 LASIK, 19 [SD1] +4 SMILE, 20 [SD 3.6] +4 reimplantation and 29%;p<0.01). Overall this represents a minimal inflammatory response with no clear increase in individual groups or at higher power of treatment, with exception of LASIK by TUNEL staining.

### Complications

One monkey suffered from microbial keratitis and one a post-operative hyphaema, which subsequently resolved.

## Discussion

Ablation based procedures such as PRK and LASIK are the currently accepted means of correcting hyperopia[6]. By implication this induces stromal thinning and steepening of the central cornea, thereby potentially affecting corneal structural integrity and stability. Currently human data shows there is no clear advantage of hyperopic PRK vs. LASIK but treatment regression has been an issue for hyperopic laser refractive procedures and risk factors include but are not confined to higher degrees of astigmatism and hyperopia [6, 30]. The results from our study give evidence that SMILE and lenticule re-implantation are alternative methods to LASIK for treating low to moderate hyperopia.

Since the advent of femtosecond laser technology, SMILE has become increasingly popular for the correction of myopia and astigmatism [13, 18, 31, 32]. This is likely attributed to the well-studied and well-established safety and efficacy profile [33–36]. Our experiments showed that in three distinct groups, there was no apparent haze, oedema or epithelial defects in any of the treatment groups as early as 1 week after surgery. There was no subjective opacity throughout the follow-up period. The refractive outcomes were comparable in the LASIK and SMILE groups and were accurate to 0.13 and 0.56D respectively at +2 and 1.4 and -0.6 D at +4 treatment, while the re-implantation group showed a regression of -2.0 D at +2 and -2.5D at +4 treatment. Whether the regression is the effect of remodelling or the depth of the implantation is not currently understood. Blum and colleagues showed that hyperopic SMILE treatment is safe and feasible in human eyes, but with regression that was inferior to myopic treatment. [27] A more recent clinical study by Sekundo et al. followed the clinical outcomes of 9 eyes, showing good predictability (within 1.0D) and stability (SE of +0.04D at 3 months), and with regression similar to LASIK procedures (0.5D). [24] Hyperopic SMILE optical zone has also been found to be larger than optical zone diameter for hyperopic LASIK. [37]

Although keratometric measurements have shown that refractive power increased, this was less than anticipated, in particular for the +4 groups. Measured at 3 months, in the +2.0D groups, the increase in refractive power ranged form 0.78 (LASIK) to 1.4 (re-implantation), with only the SMILE group approaching statistical significance (p = 0.08). In the +4.0D groups, the 3-month changes were between 0.90 (re-implantation) and 1.5 (SMILE) with only a change of 1.2 in the LASIK group approaching statistical significance (p = 0.058). The modest change in keratometry could be due to various factors, including post-surgical epithelial wound healing processes and the creation of a corneal cap. The deviation from the predicted in the re-implantation groups could also be attributed to the thickness of the created cap (160μm), limiting the implanted lenticule’s flexibility to bulge. The data would also suggest that for lower treatment powers for hyperopic implantation a more superficial implantation may be preferable. Other human studies have implanted at a depth of 100–120μm. [10, 22–24] Furthermore, unlike in human corneas, the inherent variability of young macaques corneal refractive power at baseline (and steep corneal curvature) could have also contributed to the difficulty in achieving more predictable measurements.

OCT imaging demonstrated the hyperopic treatment profiles achieved by the differing approaches. Paracentral corneal thinning was most noticeably, but expectedly increased post SMILE, with a greater effect at +4.0D treatments, due to the extraction of a concave lenticule. Pradhan et. al, have shown that lenticule re-implantation procedure restored corneal stromal volume [21] and while our data supports an expected increase in the central thickness for +2D treatment (+16μm), this was not evident at 4D treatment. Further evaluation is required to establish the effect of higher degrees of treatment and the depth of the cap on restoring volume.

In-vivo confocal imagery demonstrated a recovery timeline that was similar across the treatment groups. The corneal stromas close to the incision planes were marked by significant hyper-reflective particle infiltration at 1 week post-operation. However, this was not significant enough to cause frank corneal haziness, as all the monkeys had clear corneas on slit-lamp examination at that time point. At 1 month post-operation, the hyper-reflective cells (secondary to activated keratocytes)[26] were much less prominent, and gradually subsided with little evidence by 3 months. By the third month, hyper-reflectivity had diminished, with no differences between groups. This was consistent with other reports showing a reduction in keratocyte activation in rabbit and non-human primates that underwent LASIK, SMILE and lenticule reimplantation by 1–4 months post treatment. [26, 28] Treatment of moderate hyperopia (+4D) did show a higher level of reflectivity compared to lower (+2D) treatment for all groups.

Our immunohistochemistry results demonstrated that, compared to LASIK, the SMILE and lenticule re-implantation procedures did not incite a higher degree of wound healing reaction, or a more significant amount of inflammation, as shown by the similar levels of fibronectin and the absence CD18 expression respectively. Tenascin, an extracellular matrix protein found in the corneal epithelial cells, is expected to be seen in the central stroma following injury [38], and in all our 6 groups of treatment, was shown to be positive along the incision plane. Collagen type I is the predominant collagen type present in the cornea stroma [39]. Our results show that this collagen was present in the full thickness of all our sample corneas, and that there were no significant alterations in the corneal stroma post LASIK, SMILE or lenticule re-implantation. This indicates that collagen expression was not impacted, thereby maintaining normal corneal transparency, consistent with our observations in the slit-lamp images. The clear corneas are also supported by our negative results in α-SMA staining, a marker for myofibroblasts, the presence of which would have contributed to corneal haziness. Leukocyte integrin β2 (CD18), both an inflammatory marker and mediator of polymorphonuclear leukocyte (PMN) migration within the corneal stroma [40], was absent in all samples when stained at the third month time point. Changes in inflammatory responses in hyperopic treatment in rabbits show a higher degree of inflammation by LASIK compared to SMILE including an increase in HSP47 expression following LASIK than SMILE. [28] Similar findings were seen in this study but a more variable pattern was seen with TUNEL staining, albeit with a statistically significant yet clinically questionable difference among groups (ranging from 8.2–11.8% positive cells). This is in keeping with the other immunological and in vivo findings suggestive of a low level of inflammation at 12 weeks post procedure.

Limitations in this study include the relatively small number of primates and the difficulty in interpreting changes in corneal thickness and refractive power. This is further complicated by the relatively steep nature of the young macaques cornea as discussed. Although biomechanical properties of the human cornea are not fully understood, we now have a clearer understanding of for example greater risk of ectasia post LASIK by measuring percentage tissue ablation rather than the flap depth [41, 42] and a steep non-human primate cornea may not automatically mean that they are ectatic. While these data may not mirror the range of K values of non-ectatic human corneas, they offer a close an approximation that can be reasonably explored.

This is the first comprehensive evaluation comparing all modalities for hyperopia. This study also sought to determine the effects of these refractive treatment modalities on mild to moderate degrees of hyperopia up to +4D, as most hyperopia is below this level. Wider applications in correcting extreme hyperopia however in the context of aphakia have previously been explored and this may offer a means of restoring volume to ectatic corneas [20, 21] Challenges in maintaining efficacy from hyperopic treatments, in particular with high hypermetropia have led to the exploration of combining procedures such as LASIK with collagen cross-linking. [43] Furthermore, high-risk groups such as those with *forme fruste* keratoconus have undergone SMILE combined with collagen cross-linking. [44] Research into the applicability and predictability of RELEx in the treatment of hyperopia is still in its relative infancy, but our study suggests that SMILE is safe and effective in treating hyperopia with respect to cellular responses, with a predictability that is comparable to LASIK. Lenticule reimplantatation showed greater regression. Although intraocular approaches such as phakic IOL implantation may be an alternative option to achieve accurate refractive correction, further investigation in the clinical setting and on the effects of cross-linking are warranted and may offer further understanding of how these modalities can be used to help patients with hypermetropia or indeed presbyopia. Specifically, cross-linking the lenticule prior to reimplantation may reduce compliance and maintain its shape.

## Supporting information

S1 Fig Schematic diagram of experimental protocol. (TIFF)

S1 File Supplementary tables. (DOCX)
