# Supplementary figures and images for: Hyperopic refractive correction by LASIK, SMILE or lenticule reimplantation in a non-human primate model
Source: PLoS One. 2018 Mar 28;13(3):e0194209. doi: 10.1371/journal.pone.0194209 (PMC5874005; doi:10.1371/journal.pone.0194209)

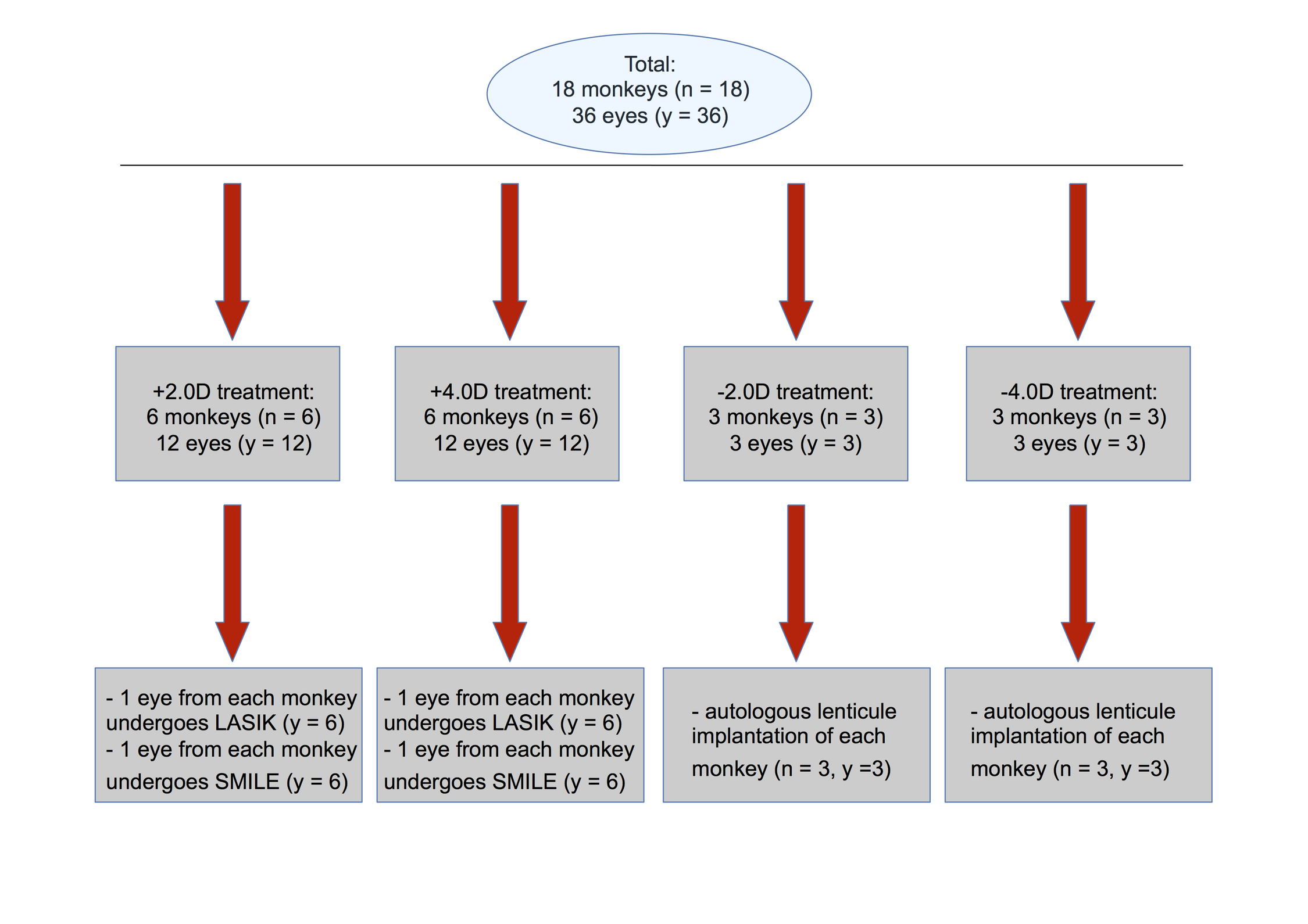

Supplement: S1 Fig — (TIFF) [file pone.0194209.s001.tiff]
