# Supplementary material for: Hyperopic refractive correction by LASIK, SMILE or lenticule reimplantation in a non-human primate model
Source: PLoS One. 2018 Mar 28;13(3):e0194209. doi: 10.1371/journal.pone.0194209 (PMC5874005; doi:10.1371/journal.pone.0194209)
Supplement: S1 File — (DOCX) [file pone.0194209.s002.docx]

**S1 File: Supplementary Tables**

| **Group** | **No.** | **Intraocular Pressure (+2D)** | | **Intraocular Pressure (+4D)** | |  | |
| --- | --- | --- | --- | --- | --- | --- | --- |
|  |  | **Time 0** | **3 months** | **Time 0** | **3 months** |  |  |
| **LASIK** | 1 | 8.0 | 6.6 | 6.4 | 7.0 |  |  |
|  | 2 | 7.8 | 5.6 | 4.6 | 3.4 |  |  |
|  | 3 | 7.2 | 7.4 | 5.6 | 4.2 |  |  |
|  | 4 | 7.0 | 6.4 | 5.0 | 3.6 |  |  |
|  | 5 | 10.6 | 5.0 | 5.2 | 5.4 |  |  |
|  | 6 | 6.2 | 4.8 | 9.0 | 6.4 |  |  |
|  |  |  |  |  |  |  |  |
| **SMILE** | 1 | 6.6 | 6.2 | 6.8 | 6.8 |  |  |
|  | 2 | 8.6 | 5.6 | 6.8 | 3.0 |  |  |
|  | 3 | 5.6 | 7.2 | 4.6 | 3.6 |  |  |
|  | 4 | 6.2 | 8.0 | 4.8 | 3.6 |  |  |
|  | 5 | 6.2 | 5.0 | 5.6 | 3.8 |  |  |
|  | 6 | 5.4 | 4.8 | 7.8 | 5.0 |  |  |
|  |  |  |  |  |  |  |  |
| **Re-implantation** | 1 | 5.4 | 7.6 | 7.8 | 6.4 |  |  |
|  | 2 | 8.0 | 10.8 | 6.6 | 8.6 |  |  |
|  | 3 | 8.0 | 8.8 | 4.8 | 13.6 |  |  |
| **Control** |  | 5.8 | 8.4 | 5.8 | 8.4 |  |  |

**Table A**

| **Group** | **No.** | **Refractive error (SE)** | | **Keratometry (k mean)** | | **Pachymetry central um (Paracentral um)** | |
| --- | --- | --- | --- | --- | --- | --- | --- |
|  |  | **Time 0** | **3 months** | **Time 0** | **3 months** | **Time 0** | **3 months** |
| **LASIK** | 1 | -0.40 | -1.25 | 58.32 | 58.21 | 390.33 (510.00) | 390.00 (489.50) |
|  | 2 | -0.77 | 0.20 | 58.87 | 61.07 | 428.33 (558.50) | 454.00 (512.17) |
|  | 3 | -1.13 | -8.16 | 60.52 | 64.91 | 373.00 (494.33) | 393.00 (474.33) |
|  | 4 | -0.46 | -3.17 | 60.50 | 59.67 | 424.33 (575.33) | 458.33 (547.50) |
|  | 5 | -0.25 | -0.63 | 58.88 | 58.65 | 355.67 (453.00) | 338.67 (459.50) |
|  | 6 | 0.83 | -1.96 | 58.85 | 58.10 | 390.00 (525.40) | 413.00 (549.20) |
|  |  |  |  |  |  |  |  |
| **SMILE** | 1 | -0.170 | -3.35 | 60.54 | 62.84 | 400.00 (518.17) | 386.67 (489.50) |
|  | 2 | -2.480 | -2.53 | 60.24 | 61.57 | 444.33 (562.33) | 408.33 (512.17) |
|  | 3 | -4.130 | -5.28 | 60.64 | 62.34 | 394.33 (504.00) | 371.33 (474.33) |
|  | 4 | -1.130 | -2.63 | 58.39 | 59.98 | 448.33 (610.00) | 431.33 (547.50) |
|  | 5 | -0.080 | -4.29 | 59.36 | 61.63 | 355.67 (465.50) | 330.67 (459.50) |
|  | 6 | -1.130 | -2.25 | 59.29 | 57.70 | 401.33 (528.17) | 401.33 (526.50) |
|  |  |  |  |  |  |  |  |
| **Re-implantation** | 1 | -0.50 | 1.13 | 58.17 | 57.53 | 495.00 (636.33) | 476.67 (667.50) |
|  | 2 | -0.25 | -0.29 | 55.60 | 58.00 | 465.33 (618.33) | 489.33 (622.33) |
|  | 3 | -0.33 | -2.04 | 56.84 | 59.35 | 434.00 (576.00) | 475.33 (582.17) |
| **Control** |  | -2.88 | 1.04 | 58.90 | 58.60 | 458.00 (605.17) | 449.67 (588.33) |

**Table B**

| **Group** | **No.** | **Refractve error (SE)** | | **Keratometry (k mean)** | | **Pachymetry central um (Paracentral um)** | |
| --- | --- | --- | --- | --- | --- | --- | --- |
|  |  | **Time 0** | **3 months** | **Time 0** | **3 months** | **Time 0** | **3 months** |
| **LASIK** | 1 | -0.58 | -4.08 | 60.07 | 61.16 | 381.67 (470.00) | 392.67 (505.83) |
|  | 2 | -2.71 | -4.54 | 60.60 | 63.44 | 401.33 (506.50) | 413.00 (528.67) |
|  | 3 | -4.17 | -3.67 | 61.99 | 61.21 | 364.33 (464.83) | 345.00 (431.17) |
|  | 4 | -0.79 | -4.92 | 56.59 | 57.62 | 404.00 (549.33) | 391.33 (523.50) |
|  | 5 | -1.50 | -5.08 | 61.77 | 62.91 | 381.67 (504.67) | 387.00 (471.50) |
|  | 6 | -1.00 | -3.83 | 57.86 | 59.63 | 419.00 (574.00) | 397.33 (519.67) |
|  |  |  |  |  |  |  |  |
| **SMILE** | 1 | -2.71 | -3.25 | 62.07 | 60.60 | 396.00 (480.17) | 377.00 (484.17) |
|  | 2 | -0.17 | -4.75 | 60.37 | 64.22 | 418.33 (552.00) | 397.33 (505.50) |
|  | 3 | -2.75 | -9.42 | 62.15 | 66.83 | 370.00 (482.50) | 336.00 (432.00) |
|  | 4 | -3.67 | -7.25 | 58.54 | 57.13 | 408.00 (533.83) | 381.67 (483.67) |
|  | 5 | -1.25 | -5.79 | 62.19 | 63.87 | 391.67 (543.83) | 359.00 (448.17) |
|  | 6 | -2.38 | 0.92 | 62.04 | 63.94 | 466.67 (613.50) | 421.33 (538.83) |
|  |  |  |  |  |  |  |  |
| **Re-implantation** | 1 | -1.79 | -3.88 | 61.87 | 63.08 | 432.67 (569.50) | 502.00 (633.67) |
|  | 2 | 0.08 | -5.63 | 59.18 | 62.23 | 481.00 (649.83) | 468.00 (611.33) |
|  | 3 | -0.88 | 2.29 | 59.65 | 58.09 | 466.67 (639.67) | 425.67 (648.67) |
| **Control** |  | -2.88 | 1.04 | 58.90 | 58.60 | 458.00 (605.17) | 449.67 (588.33) |

**Table C**

| **Group** |  | **+2 Treatment Group** | | **+4 Treatment Group** | |
| --- | --- | --- | --- | --- | --- |
|  |  | **HSP 47**  **(**+ve cells/field**)** | **TUNEL**  **(%)** | **HSP 47**  **(**+ve cells/field**)** | **TUNEL**  **(%)** |
| **LASIK** | 1 | 36 | 8.2 | 39 | 10.6 |
|  | 2 | 33 | 9.0 | 39 | 12.6 |
|  | 3 | 30 | 8.7 | 36 | 12.1 |
| Mean (SD) |  | 33 (3) | 8.6 (0.4) | 38 (1.7) | 8.6 (0.4) |
|  |  |  |  |  |  |
| **SMILE** | 1 | 18 | 10.5 | 20 | 10.0 |
|  | 2 | 16 | 11.6 | 18 | 9.7 |
|  | 3 | 18 | 10.6 | 20 | 9.9 |
| Mean (SD) |  | 17.3 (1.2) | 10.9 (0.6) | 19.3 (1.2) | 10.9 (0.6) |
|  |  |  |  |  |  |
| **Re-implantation** | 1 | 18 | 8.3 | 17 | 9.7 |
|  | 2 | 20 | 8.4 | 24 | 9.5 |
|  | 3 | 18 | 7.9 | 19 | 9.6 |
| Mean (SD) |  | 18.7 (1.2) | 8.2 (0.3) | 20 (3.6) | 9.6 (0.1) |

**Table D**
